# Extracellular Traps in Coronary Thrombus Aspirates from Patients with ST-Elevation Myocardial Infarction

**DOI:** 10.3390/ijms27135998

**Published:** 2026-07-03

**Authors:** Dalia Pangonytė, Sandrita Šimonytė, Vaiva Lesauskaitė, Vitalija Siratavičiūtė, Giedrė Bakšytė, Jolanta Marcinkevičienė, Zita Stanionienė, Lina Utkienė, Lina Jusienė, Reda Radikė, Gediminas Jaruševičius, Ramūnas Unikas, Astra Vitkauskienė, Olivija Dobilienė

**Affiliations:** 1 Laboratory of Cardiac Pathology, Institute of Cardiology, Lithuanian University of Health Sciences, 50162 Kaunas, Lithuania; vaiva.lesauskaite@lsmu.lt (V.L.); vitalija.sirataviciute@lsmu.lt (V.S.); jolanta.elena.marcinkeviciene@lsmu.lt (J.M.); zita.stanioniene@lsmu.lt (Z.S.); lina.utkiene@lsmu.lt (L.U.); lina.jusiene@lsmu.lt (L.J.); reda.radike@lsmu.lt (R.R.); 2 Laboratory of Molecular Cardiology, Institute of Cardiology, Lithuanian University of Health Sciences, 50162 Kaunas, Lithuania; sandrita.simonyte@lsmu.lt; 3 Heart Centre, Lithuanian University of Health Sciences, 50161 Kaunas, Lithuania; giedre.baksyte@lsmu.lt (G.B.); ramunas.unikas@lsmu.lt (R.U.); olivija.dobiliene@lsmu.lt (O.D.); 4 Laboratory of Automation of Cardiovascular Investigation, Institute of Cardiology, Lithuanian University of Health Sciences, 50162 Kaunas, Lithuania; gediminas.jarusevicius@lsmu.lt; 5 Department of Laboratory Medicine, Lithuanian University of Health Sciences, 50161 Kaunas, Lithuania; astra.vitkauskiene@lsmu.lt

**Keywords:** extracellular traps, coronary thrombus, ST-elevation myocardial infarction

## Abstract

The formation of extracellular traps (ETs) through ETosis has emerged as a key mechanism in immunothrombosis. However, the temporal dynamics and clinical significance of ETosis in coronary thrombi of ST-elevation myocardial infarction (STEMI) patients remain incompletely understood. We investigated whether ETosis burden increases with thrombus age and is associated with *DNASE1* and *TREX1* genetic variants as well as impaired myocardial reperfusion. Thrombus aspirates from 81 STEMI patients undergoing primary percutaneous coronary intervention were histologically classified as fresh (*n* = 41) or lytic (*n* = 40). ETosis was quantified by citrullinated histone H3 (CitH3) immunohistochemistry and digital image analysis, complemented by multiplex staining for myeloperoxidase (MPO), CD68, caspase 3, and CD61. Plasma ET-related markers and genotyping of *DNASE1* (rs1053874) and *TREX1* (rs11797) were also performed. CitH3-positive cells were present in all thrombi but were more abundant in lytic (older) thrombi compared with fresh thrombi (1348 vs. 591 cells/mm^2^, *p* < 0.001). Increased ETosis was associated with neutrophil and macrophage infiltration, apoptosis, prolonged ischemia time, elevated systemic inflammation (neutrophil–lymphocyte ratio and C-reactive protein), and impaired myocardial reperfusion (lower TIMI flow grades). Moreover, the *DNASE1* GG genotype was associated with higher densities of MPO- and CD68-positive cells, whereas the *TREX1* CC genotype was associated with increased densities of CitH3-, MPO-, and CD68-positive cells. This study demonstrates that ETosis increases with coronary thrombus maturation and is associated with local inflammation and impaired reperfusion in STEMI. Genetic variants in *DNASE1* and *TREX1* may modulate inflammatory cell accumulation within thrombi. These findings suggest ETosis as a potential therapeutic target, particularly in patients with delayed presentation.

## 1. Introduction

Acute ST-segment elevation myocardial infarction (STEMI) remains a leading cause of cardiovascular mortality and morbidity on a worldwide scale. Ischemic heart disease, of which STEMI constitutes a major acute manifestation, accounts for millions of deaths annually and imposes a tremendous burden on healthcare systems [1,2]. Despite considerable advances in primary prevention, rapid diagnosis, and reperfusion therapy, the abrupt occlusion of a coronary artery by a thrombotic mass remains the central pathophysiological event leading to myocardial infarction [3].

In the past two decades, timely primary percutaneous coronary intervention (pPCI) with stent implantation has significantly enhanced short-term survival and reduced the incidence of mechanical complications, including ventricular septal and free wall rupture [4]. Nevertheless, 30–40% of patients still achieve suboptimal myocardial reperfusion, as reflected by incomplete ST-segment resolution, reduced Thrombolysis in Myocardial Infarction (TIMI) flow grades, and the presence of microvascular obstruction. These phenomena are associated with larger final infarct size, impaired left ventricular systolic function, adverse ventricular remodelling, and significantly worse long-term prognosis [5]. Such observations underscore the pressing need for a more comprehensive understanding of the cellular and molecular mechanisms that regulate thrombus formation, growth, stabilisation, and eventual resolution at the site of the culprit lesion.

Thrombus aspiration provides direct and immediate access to fresh thrombotic material from the site of coronary occlusion during emergency pPCI [6]. Consequently, the histological, immunohistochemical and molecular analyses of aspirated thrombi have become as a robust platform for translational research, providing unparalleled insights into the in vivo processes of human coronary thrombogenesis.

For an extended period, coronary thrombosis was conceptualised almost exclusively through the lens of platelet activation and the coagulation cascade triggered by exposure of subendothelial collagen and tissue factor following atherosclerotic plaque rupture. However, a growing body of evidence has led to a shift in the prevailing paradigm towards an integrated model of immunothrombosis, in which innate immune cells and inflammatory pathways play critical roles in both the initiation and propagation of intracoronary thrombi [7,8,9,10,11].

In this framework, the formation of extracellular traps (ETs) via a specialised form of programmed cell death known as ETosis has emerged as one of the most significant and intriguing mechanisms [12,13,14]. Initially described in neutrophils as neutrophil extracellular trap (NET) formation, ETosis is now recognised as a broader, multicellular process that also involves monocytes/macrophages, eosinophils, and mast cells [15,16,17]. The process is orchestrated by peptidylarginine deiminase 4 (PAD4)-mediated citrullination of histones, leading to chromatin decondensation, nuclear envelope breakdown, and the release of web-like structures composed of decondensed DNA, citrullinated histone H3 (CitH3), myeloperoxidase (MPO), neutrophil elastase (NE), and other granular proteins [16,17,18].

ETs initially evolved as a host defence mechanism to trap and destroy pathogens. However, in aseptic inflammatory conditions, such as acute coronary syndromes, these structures have been shown to exert potent prothrombotic and proinflammatory effects. ETs have been demonstrated to provide a structural scaffold that promotes platelet adhesion and aggregation, facilitates the assembly and activation of coagulation factors, upregulates tissue factor expression on endothelial cells and monocytes, induces endothelial dysfunction, and directly contributes to microvascular obstruction and the clinically detrimental “no-reflow” phenomenon [10,17,18,19,20,21,22].

As indicated by earlier research, the presence of ETs has been confirmed in coronary thrombi of patients with STEMI. Mangold et al. [23] demonstrated abundant ETs at the culprit lesion and a significant negative correlation with post-PCI ST-segment resolution. Blasco et al. [24] detected ETs in approximately 51% of thrombus aspirates and established a correlation between higher ET burden and increased risk of major adverse cardiac events. Further studies have associated elevated circulating ET levels with poorer microvascular perfusion, larger infarct size, and adverse left ventricular remodelling [21,25].

Coronary thrombi are highly dynamic lesions whose composition evolves over time. Standardised histological classification systems can reliably distinguish “fresh” thrombi, characterised by well-preserved platelets, erythrocytes, and intact granulocytes, from “lytic” (older) thrombi showing features of cell destruction, cell disintegration, macrophage infiltration, foci of necrosis, and extensive fibrin deposition [26,27,28]. Emerging evidence suggests that ETosis may be the predominant mode of cell death during thrombus maturation [29]. However, robust quantitative data comparing ET burden across different thrombus ages, together with their relationship to systemic inflammation, duration of ischemia, and myocardial reperfusion success, remain scarce. Furthermore, there is a paucity of research focusing on the relationship between ETs and non-neutrophil cells.

The persistence of ETs is subject to stringent regulation by extracellular DNA-degrading enzymes deoxyribonuclease 1 (DNase I) and three-prime repair exonuclease 1 (TREX1). Although common functional polymorphisms in *DNASE1* (rs1053874) and *TREX1* (rs11797) have been shown to impair DNA degradation and are associated with increased thrombotic risk and worse outcomes after myocardial infarction, their direct influence on the cellular composition and ET burden of human coronary thrombi has never been investigated in vivo [30,31,32].

While previous studies have demonstrated the prevalence of ETs in STEMI thrombi, their inverse relationship with microvascular perfusion, and the potential role of ETosis in thrombus maturation, several significant gaps remain. Specifically, there has been no systematic investigation of the following: robust quantitative data on the ET burden across thrombi of different ages (particularly fresh and lytic thrombi); the contribution of non-neutrophil cells to ETosis in coronary thrombi; and the in vivo impact of *DNASE1* and *TREX1* polymorphisms on the cellular composition of thrombi. This study addresses these gaps by using a standardised thrombus age classification system, performing multiplex immunohistochemistry with digital quantification, profiling plasma biomarkers, and genotyping candidate genes in a well-characterised cohort of STEMI patients.

## 2. Results

A total of 81 patients with STEMI undergoing pPCI with thrombus aspiration were included. The mean age was 64.19 ± 13.1 years, and 71.6% were male. The median time from symptoms onset to pPCI was 3.70 h (IQR: 2.56–10.48). Complete perfusion (TIMI flow grade 3) was achieved in 83.95% of cases. In-hospital mortality occurred in 6 patients (7.4%).

### 2.1. Thrombus Aspirate Histological Composition

**Figure 1:**
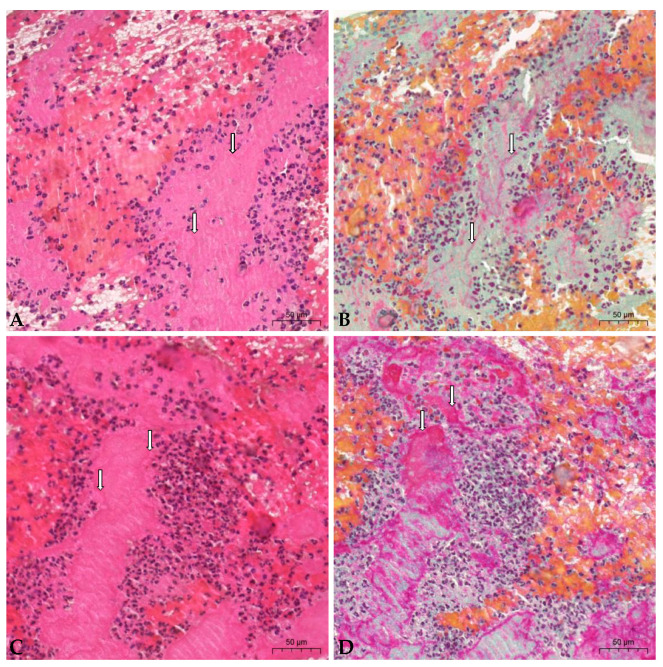
Representative histological images of coronary artery material extracted via thrombus aspiration from patients with ST-segment elevation myocardial infarction. (**A**,**B**) Fresh thrombus containing intact blood cells, including granulocytes (dark blue nucleus in both stains), platelets (pink in HE and turquoise in Picro-Mallory stain), erythrocytes (red in HE and yellow in Picro-Mallory stain), and fibrin (arrow). (**C**,**D**) Lytic thrombus with cellular degradation. (**A**,**C**) HE stain; (**B**,**D**) Picro-Mallory stain. Scale bar: 50 μm. Images were captured from digital slides with an original magnification of 20×. Abbreviations: HE, hematoxylin and eosin stain.

A histological evaluation of hematoxylin and eosin (HE) and Picro-Mallory stained sections revealed distinct features according to thrombus age. Fresh thrombi (*n* = 41) demonstrated layered aggregates of platelets, erythrocytes, intact neutrophils, and fibrin strands (Figure 1A,B). Lytic thrombi (*n* = 40) exhibited cellular degradation, including karyorrhexis and karyolysis of neutrophils, abundant fibrin deposition, foci of necrosis, macrophage infiltration, and granulocytes showing polarisation and lytic activity towards fibrin and platelet surfaces (Figure 1C,D). In five cases, thrombi exhibited signs of organisation, yet the area affected was less than 5% of the total thrombus area, and no further evaluation was conducted on this area.

Atherosclerotic plaque components were identified in 23 aspirates (28.4%), including extracellular lipids, CD68-positive macrophages (foam cells), cholesterol crystals, fibroelastic tissue (Figure S1), as well as calcium deposits, smooth muscle cells (α-SMA-positive), and endothelial cells (CD34-positive).

### 2.2. Thrombus Immunohistochemical Components

**Figure 2:**
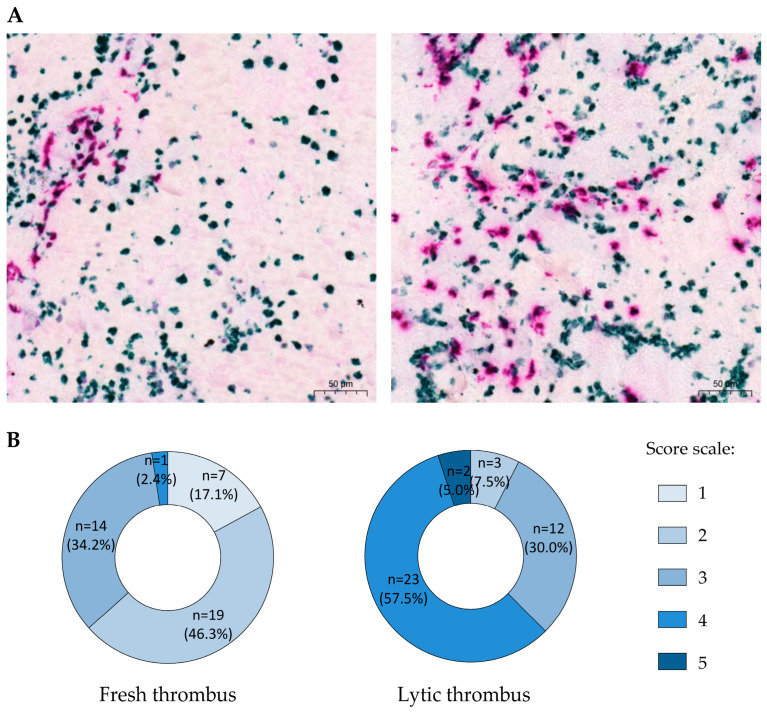
Expression patterns of ETs. (**A**) Representative images double-staining immunohistochemistry: CitH3 (red) overlaps with MPO (green) in fresh (**left**) and lytic (**right**) thrombi. Scale bar: 50 μm. Images were captured from digital slides with an original magnification of 20×. (**B**) Pie charts depict group stratification according to the CitH3 score scale. Abbreviations: MPO, myeloperoxidase; CitH3, citrullinated histone H3.

Immunohistochemistry revealed the presence of CitH3-positive cells (a widely used marker of ETosis) in all thrombus aspirates. However, the extent of CitH3 positivity differed markedly between groups when quantified relative to MPO-positive cells in double-stained slides (Figure 2). In fresh thrombi, the proportion of cells that were both CitH3-positive and MPO-positive was relatively small: score 1 (less than 1%) in approximately one-fifth of samples, score 2 (1–10%) in nearly half of the samples, and score 3 (11–33%) in approximately one-third of the samples. In lytic thrombi, however, CitH3 positivity was substantially higher, with slightly less than one-third of samples being assigned score 3 and more than half showing score 4 (34–66% of CitH3-positive cells among MPO-positive cells).

**Figure 3:**
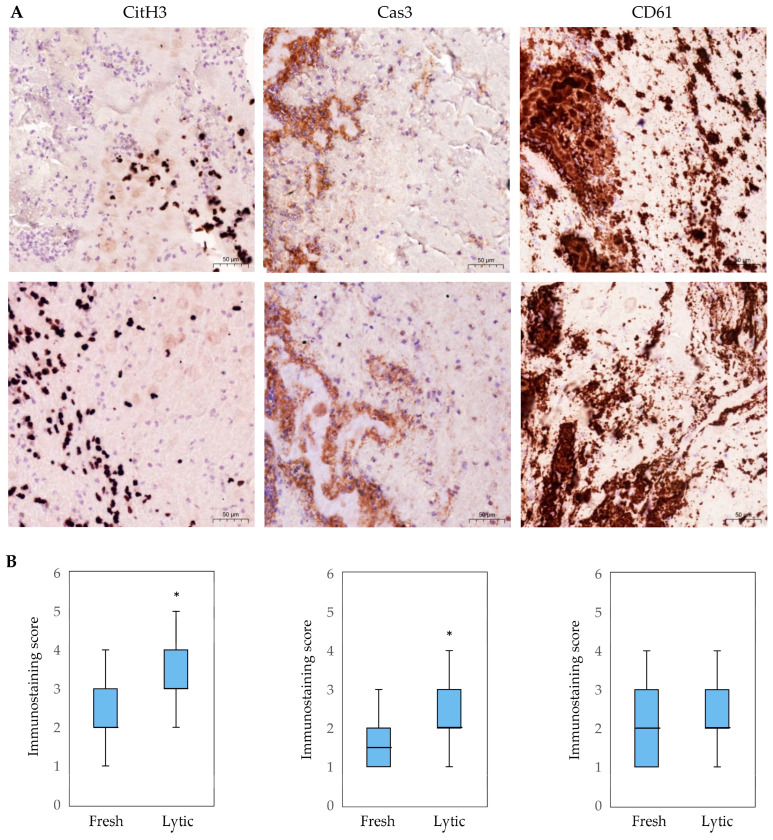
Expression patterns of immunohistochemical staining. (**A**) Comparative expression of CitH3, Cas3, and CD61 in fresh (upper panel) and lytic (lower panel) thrombus. Stain with DAB chromogen (brown). Scale bar: 50 μm. Images were captured from digital slides with an original magnification of 20×. (**B**) Semi-quantitative immunohistochemical analysis; data are presented as median, the 25th and 75th percentiles, and minimum and maximum values (whiskers); the Mann–Whitney U test was used, * *p* < 0.001 for lytic (*n* = 40) vs. fresh thrombus (*n* = 41) groups. Abbreviations: Cas3, caspase 3; CD, cluster of differentiation; CitH3, citrullinated histone H3.

Semi-quantitative analysis revealed an association between the expression of CitH3, Cas3, and CD61 and thrombus histological age. The median immunostaining scores for the CitH3 and Cas3 markers were found to be significantly higher in lytic samples compared with fresh thrombi (*p* < 0.001 for each; Figure 3). As illustrated in Figure S2, cells that were positive for CitH3 and caspase 3 (Cas3, an apoptosis marker) were frequently localised in close proximity to CD61-positive platelets. The presence of Cas3 staining was observed in both nucleated cells (predominantly granulocytes) and platelets.

**Figure 4:**
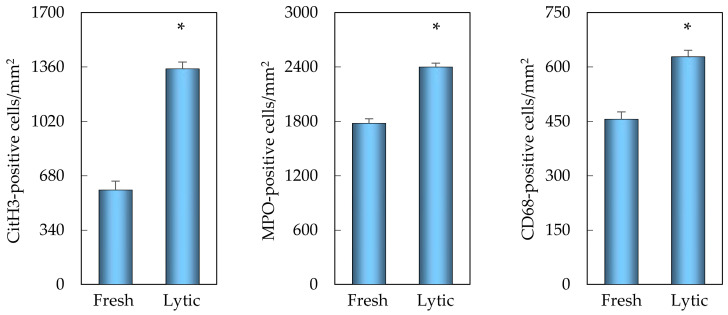
Quantification of CitH3-, MPO-, and CD68-positive cells in fresh (*n* = 41) and lytic (*n* = 40) thrombus groups. Digital image analysis of DAB stain. Bars represent estimated marginal means and standard errors derived from a linear mixed-effects model. * *p* < 0.001 for lytic vs. fresh thrombus groups. Abbreviations: CD, cluster of differentiation; MPO, myeloperoxidase; CitH3, citrullinated histone H3.

Quantitative digital image analysis of DAB-stained sections (Figure S3) revealed that the density of CitH3-positive cells was significantly higher in lytic thrombi compared to fresh thrombi (mean 1348 vs. 591 cells/mm^2^, *p* < 0.001; Figure 4). The density of MPO-positive (a marker of neutrophils and monocytes/macrophages) cells in lytic thrombi was 1.3-fold higher than in fresh thrombi (mean 2399 vs. 1779 cells/mm^2^, *p* < 0.001). In a similar manner, the density of CD68-positive (a marker of monocytes/macrophages) cells was found to be 1.4-fold higher in lytic thrombi in comparison to fresh thrombi (mean 628 vs. 457 cells/mm^2^, *p* < 0.001). It is noteworthy that the proportion of CD68- and MPO-positive cells remained constant in both groups. In addition, a proportion of the CD68-positive cells were also positive for the CitH3 marker (Figure S4).

In addition, the presence of zones containing CD68, MPO, and CitH3-positive cells was observed in atherosclerotic plaques (see Figure S1).

In fresh thrombi, the CitH3 immunostaining score exhibited a positive correlation with the density of CitH3-positive cells (Spearman’s ρ = 0.693, *p* < 0.01). The present study has revealed a comparable correlation between these two parameters and the Cas3 immunostaining score (ρ = 0.500, *p* < 0.01 and ρ = 0.561, *p* < 0.01, respectively) and with the density of MPO-positive cells (ρ = 0.444, *p* < 0.01 and ρ = 0.548, *p* < 0.01, respectively; Table 1). A positive correlation was observed between MPO- and CD68-positive cell densities (ρ = 0.592, *p* < 0.01). A further correlation was identified between CD68-positive cells and the CD61 immunostaining score (ρ = 0.417, *p* < 0.05).

In lytic thrombi, a correlation has been identified between CitH3 immunostaining score and CitH3-positive cell density (ρ = 0.662, *p* < 0.01) as well as between CitH3-positive cell density and Cas3 immunostaining score (ρ = 0.364, *p* < 0.05). Additionally, a positive correlation has been identified between CitH3- and CD68-positive cell densities (ρ = 0.558, *p* < 0.01) as well as between CD61 immunostaining score and CD68-positive cell density (ρ = 0.406, *p* < 0.05).

### 2.3. Association of Plasma ET-Related Markers, Clinical and Laboratory Variables with Thrombus Components

Plasma median levels of ETosis-related markers (i.e., CitH3, double-stranded DNA (dsDNA), MPO and NE were observed to be elevated in the lytic compared to the fresh thrombus group. However, these differences did not attain statistical significance (Figure S5).

The presence of lytic thrombi was found to be associated with a prolonged time from symptoms onset to pPCI (median 5.29 h [IQR 3.08–13.52] vs. 3.38 h [2.27–8.56], *p* < 0.05), a lower lymphocyte count (*p* < 0.05), an elevated neutrophil-to-lymphocyte ratio (NLR, *p* < 0.01), and elevated C-reactive protein (*p* < 0.05) levels (Table 2). No significant differences were observed in age, sex, smoking, hypertension, white blood cell or neutrophil counts, platelet count, coagulation parameters, or high-sensitivity troponin I levels. The distribution of the infarct-related artery and post-procedural TIMI flow grades also did not differ significantly between groups.

In fresh thrombi, there was a moderate correlation between plasma NE concentration and CitH3-positive cell density (ρ = 0.546, *p* < 0.01; Table 3). Furthermore, positive correlations were identified between CitH3 density and the following parameters: white blood cell count (ρ = 0.382, *p* < 0.05), neutrophil count (ρ = 0.411, *p* < 0.05), and NLR (ρ = 0.386, *p* < 0.05). No significant correlations were observed with time from symptoms onset to pPCI or other laboratory variables for other immunohistochemical parameters.

In lytic thrombi, plasma MPO levels correlated with the Cas3 immunostaining score (ρ = 0.463, *p* < 0.01, see Table 3). Furthermore, a positive correlation was observed between white blood cell and neutrophil counts and MPO-positive cell density (ρ = 0.444, and 0.484, respectively, both *p* < 0.01) and Cas3 immunostaining score (ρ = 0.445 and 0.410, respectively, both *p* < 0.01). NLR (ρ = 0.434, *p* < 0.01) and C-reactive protein (ρ = 0.492, *p* < 0.01) levels were also correlated with MPO-positive cells. It is noteworthy that an increased time from symptoms onset to pPCI correlated with elevated MPO-positive cell density (ρ = 0.536, *p* < 0.01).

**Figure 5:**
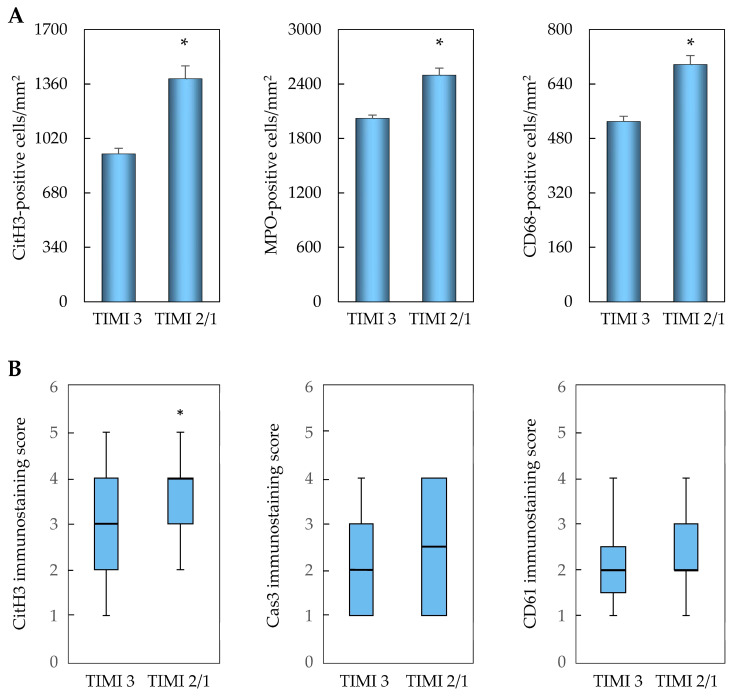
Quantification of thrombus components by TIMI group. (**A**) Digital image analysis of DAB stain. Bars represent estimated marginal means and standard errors derived from a linear mixed-effects model accounting for thrombus histological age (fresh vs. lytic). (**B**) Semi-quantitative immunohistochemical analysis: data are presented as median, the 25th and 75th percentiles, and minimum and maximum values (whiskers); the Mann–Whitney U test was used. * *p* < 0.001. Abbreviations: Cas3, caspase 3; CD, cluster of differentiation; CitH3, citrullinated histone H3; MPO, myeloperoxidase; TIMI, Thrombolysis in Myocardial Infarction flow grade.

Thrombi associated with reduced myocardial perfusion (lower TIMI flow grades) following pPCI exhibited significantly higher densities and scores of CitH3, as well as densities of MPO- and CD68-positive cells (*p* < 0.001; Figure 5). This association was observed across the entire cohort (both fresh and lytic thrombi) and derived from linear mixed-effects models accounting for thrombus histological age (fresh vs. lytic) as a grouping factor.

### 2.4. Association of DNASE1 Gene (rs1053874) and TREX1 Gene (rs11797) Polymorphisms with Thrombus Components

**Figure 6:**
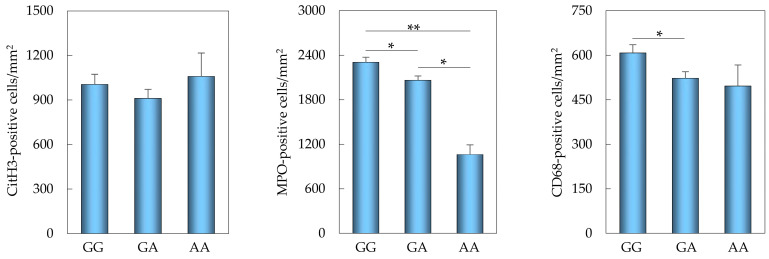
Quantification of CitH3, MPO, and CD68 according to the SNP of *DNASE1* gene (rs1053874). Digital image analysis of DAB stain. Bars represent estimated marginal means and standard errors derived from a linear mixed-effects model with Bonferroni correction. * *p* < 0.05, ** *p* < 0.001. Abbreviations: CD, cluster of differentiation; MPO, myeloperoxidase; CitH3, citrullinated histone H3; SNP, single-nucleotide polymorphism.

**Figure 7:**
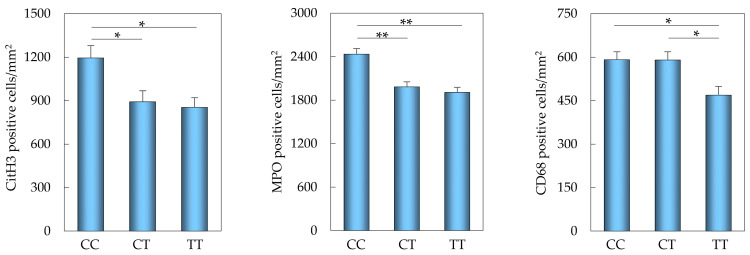
Quantification of CitH3, MPO, and CD68 according to the SNP of *TREX1* gene (rs11797). Digital image analysis of DAB stain. Bars represent estimated marginal means and standard errors derived from a linear mixed-effects model with Bonferroni correction. * *p* < 0.05, ** *p* < 0.001. Abbreviations: CD, cluster of differentiation; MPO, myeloperoxidase; CitH3, citrullinated histone H3, SNP, single-nucleotide polymorphism.

Genotyping revealed associations between specific single nucleotide polymorphisms (SNP) and thrombus cellular composition. For the *DNASE1* rs1053874 polymorphism, GG genotype carriers (compared with GA/AA) showed significantly higher densities of MPO- and CD68-positive cells (Figure 6). For the *TREX1* rs11797 polymorphism, CC genotype carriers (compared with CT/TT) were associated with higher densities of CitH3-, MPO-, and CD68-positive cells (Figure 7).

In summary, the presence of ETosis (as indicated by CitH3 positivity) was observed in all coronary thrombi, with lytic (older) thrombi exhibiting significantly more pronounced ETosis. This process was closely linked to neutrophil and monocyte/macrophage involvement and apoptosis, longer time from symptoms onset to pPCI, heightened systemic inflammation (elevated NLR and C-reactive protein), and impaired vascular reperfusion after pPCI. Genetic polymorphisms in *DNASE1* and *TREX1*, which encode enzymes involved in DNA degradation, appeared to modulate the extent of ETosis and inflammatory cell accumulation within the thrombi. These findings emphasise the dynamic evolution of coronary thrombi in STEMI and imply that ETosis may play a pivotal role in thrombus progression and clinical outcomes.

## 3. Discussion

The present study shows that ETosis burden, as measured by CitH3-positive cell density, increases substantially during coronary thrombus maturation from fresh to lytic stages (mean 591 vs. 1348 cells/mm^2^, *p* < 0.001). This increase is associated with parallel rises in MPO-positive and CD68-positive inflammatory cells as well as Cas3 positivity. These findings lend support to the concept that ETosis constitutes an important component of immunothrombosis that evolves over time within the coronary thrombus [27,29].

Traditionally, coronary thrombosis has been viewed primarily as a process driven by platelet activation and the coagulation cascade. However, over the past decade, an accumulation of evidence has emerged to support a more extensive immunothrombosis paradigm, in which innate immune mechanisms and inflammation play a substantial role in both thrombus initiation and propagation [7,8,10]. The formation of ETs through a specialised form of programmed cell death termed ETosis is central to this concept. During the process of ETosis, a range of cells, including neutrophils, monocytes/macrophages, and others, release decondensed chromatin webs that are decorated with CitH3, MPO, NE, and other granular proteins [15,17,18,33]. These web-like structures have been shown to act as a potent prothrombotic scaffold, directly interacting with platelets via histones and surface receptors. They have also been demonstrated to promote platelet adhesion and aggregation, facilitate the assembly and activation of coagulation factors [21,22,34].

The histological classification of coronary thrombi into fresh and lytic stages revealed a clear time-dependent increase of ETosis in the present study. In fresh thrombi, ETotic activity was found to be relatively limited, with CitH3-positive cells constituting less than 10% of MPO-positive cells in the majority of samples. This finding reflects an early, contained response to plaque rupture and initial thrombus formation. In contrast, lytic (older) thrombi exhibited substantially higher ET burden, with more than half of the samples showing 34–66% CitH3-positive cells among MPO-positive cells. This was accompanied by parallel increases in MPO-positive and CD68-positive cell densities, as well as more pronounced apoptosis (Cas3 positivity). This finding indicates that ET formation intensifies during thrombus ageing, potentially contributing to thrombus stabilisation, fibrin deposition and inflammatory amplification.

The present study constitutes a substantial extension of the findings of earlier histopathological investigations. Mangold et al. [23] demonstrated abundant neutrophil extracellular traps at the culprit lesion site in patients with STEMI and established a correlation between higher coronary ET burden and impaired ST-segment resolution after PCI. Blasco et al. [24] detected ETs in approximately 51% of thrombus aspirates. The authors concluded that a higher ETs load was associated with an increased risk of major adverse cardiac events, including early stent thrombosis. Pertiwi et al. [15,29] were among the first to report that ETosis represents the predominant mode of cell death during coronary thrombus maturation. The ETosis rate was found to be significantly higher in lytic and fresh thrombi than in late organised thrombi. The findings of Pituk et al. [35] exhibited a congruent outcome. Our investigation provides robust quantitative digital analysis confirming a cell density of CitH3-positive cells that is more than twice as high in lytic thrombi as in fresh thrombi. It also provides a simultaneous assessment of the contribution of MPO-positive cells (which stain neutrophils and some monocytes/macrophages) and CD68-positive cells (which stain monocytes/macrophages).

These immunohistochemical findings reveal a complex, interdependent mechanistic network linking ETosis, classical apoptosis, platelet activation, and monocyte/macrophage involvement within the coronary thrombus microenvironment. CitH3-positive cells (a marker of ETosis) and Cas3-positive cells (a hallmark of apoptosis) frequently colocalised in the same microscopic fields, often in close spatial proximity to CD61-positive platelets. This colocalisation may support the hypothesis that ETosis and apoptosis operate synergistically during thrombus maturation [18,34,36,37]. Such convergence may occur through several overlapping molecular pathways. Histone citrullination by PAD4, a pivotal step in ETosis, has been shown to promote chromatin decondensation, thus facilitating both suicidal ETosis and apoptotic nuclear fragmentation. Conversely, the release of DNA during secondary ET-like processes can be observed in apoptotic cells in cases where clearance is impaired. The frequent presence of Cas3 positivity not only in granulocytes but also in platelets themselves further supports bidirectional crosstalk: activated platelets can trigger ETs formation via P-selectin and high-mobility group box 1 signalling, while ET-derived histones and DNA can, in turn, activate platelets and promote thrombin generation [10,12,13,19,38,39,40,41,42]. Of particular significance is the demonstration, provided by the data, of partial overlap between CitH3, MPO, and CD68 positivity. This finding indicates that monocytes/macrophages within the thrombus also contribute to extracellular trap formation (so-called METs) [14,15,16]. Furthermore, CD68-positive macrophages were found to be 1.4 times more abundant in lytic thrombi than in fresh thrombi. The proportion of CD68- and MPO-positive cells remained constant in both groups. It is hypothesised that these macrophages amplify the ETotic cascade by releasing additional proinflammatory mediators, phagocytosing partially degraded cells, and providing a sustained source of tissue factor and cytokines [14,16]. The aggregate of these interactions gives rise to a potential self-amplifying loop of immunothrombosis. The process of ETosis has been shown to provide a DNA-histone scaffold that promotes platelet adhesion and aggregation. The subsequent activation of platelets has been demonstrated to enhance further ETs release. Apoptotic cells and uncleared ETs have been observed to recruit and activate monocytes/macrophages. The resulting inflammatory milieu has been shown to drive thrombus aging from the fresh to the lytic stage [29,34]. This integrated network provides a rationale for the association observed between higher densities of CitH3-, MPO-, and CD68-positive cells and impaired post-procedural myocardial reperfusion (lower TIMI flow grades, *p* < 0.001).

Plasma levels of ET-related markers (CitH3, dsDNA, MPO, NE; see Figure S3) exhibited a tendency to increase in the lytic thrombus group; however, this increase did not reach statistical significance. These findings are consistent with the extant literature, which indicates a dissociation between intense local ETotic activity within the coronary thrombus and systemic biomarker levels [20,43,44,45,46]. On the other hand, a positive correlation between plasma NE concentration and CitH3-positive cell density, observed in fresh thrombi (ρ = 0.546, *p* < 0.01), may suggest that circulating ET-related biomarkers may predominantly capture early ETotic activity. In more advanced lytic stages, local tissue remodelling and ET persistence predominate. It is important to recognise that circulating levels of ET-related markers may be associated with other processes during which extracellular traps are formed in the body. The formation of a thrombus at a ruptured atherosclerotic plaque in the coronary arteries occurs in the presence of a substantial accumulation of CitH3-positive macrophages (foam cells) within the plaque itself [8,9,15,47,48]. Conversely, coronary artery obstruction due to thrombus results in the necrosis of cardiomyocytes (myocardial infarction). The presence of neutrophils that have undergone ETosis has been observed in this area [8,49,50]. Consequently, the level of circulating ET-related markers may be associated with concurrent biological processes [51]. For instance, an increase in DNase I activity of a significant magnitude has been observed in the initial three hours following myocardial infarction [23]. Furthermore, a negative correlation has been demonstrated between DNase I activity and ET burden in the coronary thrombus [52].

This apparent discrepancy between the marked local increase in CitH3-positive cell density (>2-fold) and the non-significant differences in plasma ET markers does not contradict, but rather improves and deepens our understanding of ETosis in the context of STEMI. The local environment of the coronary thrombus appears to favour the retention and persistence of ET structures, while systemic circulation is subject to rapid clearance mechanisms and dilution from multiple sources. This compartmentalisation underscores the superior sensitivity of direct histopathological and quantitative digital image analysis (QuPath) in detecting site-specific immunothrombotic processes, in comparison to circulating surrogates. Concurrently, it elucidates the rationale underlying the utility of plasma markers in prognostic stratification, though these markers may not always accurately reflect the intensity of ETosis at the culprit lesion.

Despite the current lack of clarity regarding the correlation between the levels of ETs in thrombi and peripheral blood, circulating ETs have been associated with clinical outcomes in patients with STEMI. Elevated plasma levels of dsDNA and other ETs components at admission or following revascularisation have been shown to be independent predictors of worse prognosis, including larger infarct size, poorer ST-segment resolution, no-reflow phenomenon, and increased long-term mortality [23,24,43,44]. Consequently, the presence of circulating ETs markers can serve as useful, non-invasive indicators of systemic thromboinflammatory burden, thereby helping to identify patients at higher risk of adverse outcomes.

The present study has revealed an association between enhanced ETosis within the coronary thrombus and systemic inflammatory markers. Lytic (older) thrombi were characterised by significantly elevated admission NLR and C-reactive protein levels, together with higher densities of CitH3-positive, MPO-positive, and CD68-positive cells compared with fresh thrombi. In fresh thrombi, CitH3-positive cell density demonstrated a positive correlation with circulating white blood cell count, neutrophil count, and NLR. In lytic thrombi, MPO-positive cell density exhibited a strong correlation with neutrophil count, NLR, and C-reactive protein. These observations align with and extend previous reports linking systemic inflammation to ET burden in STEMI. Elevated NLR and C-reactive protein have been consistently associated with adverse outcomes in acute myocardial infarction and reflect heightened innate immune activation that promotes ETosis [23,25]. Studies examining coronary aspirates have demonstrated that increased local ET components (including CitH3 and MPO) are associated with systemic inflammatory markers and predict larger infarct size, microvascular obstruction, and poorer ST-segment resolution [24,46]. In addition, persistent local ETosis in ageing thrombi has been shown to sustain thrombo-inflammation through continuous recruitment of neutrophils and macrophages, forming a self-amplifying loop [15,25]. These data underscore the systemic inflammatory indices (e.g., NLR and C-reactive protein) not only reflecting but actively contributing to the progression of ET-driven immunothrombosis at the culprit lesion.

Although CitH3 is one of the most widely adopted immunohistochemical markers for ETosis in human tissues, including coronary thrombi [23,24,46], we acknowledge important limitations regarding its specificity. Histone citrullination, catalysed by PAD4, is not exclusive to ETosis. It can also occur during apoptosis, where calcium influx activates PAD enzymes, leading to the citrullination of histones and cytoskeletal proteins. This facilitates nuclear condensation and the formation of apoptotic bodies, independent of ET release, but only if efferocytosis mechanisms fail [53].

Nevertheless, there are several lines of evidence that support our interpretation that the markedly increased CitH3 density in lytic thrombi primarily indicates enhanced ETosis during thrombus maturation. Firstly, the number of CitH3-positive cells was significantly higher in older lytic thrombi (1348 vs. 591 cells/mm^2^, *p* < 0.001). These thrombi are characterised by cellular degradation, macrophage infiltration, and fibrin reorganisation—conditions that favour ETosis. Secondly, CitH3 density showed a strong correlation with MPO- and CD68-positive cell densities, as well as with plasma NE. Thirdly, elevated CitH3 was clinically associated with prolonged ischaemic time, heightened systemic inflammation and impaired myocardial reperfusion—outcomes that are consistently associated with immunothrombosis driven by extracellular traps in the literature [23,24]. Furthermore, no overlap has been demonstrated between the CitH3 and Cas3 markers until now. Thus, within this multimodal context, CitH3 provides evidence in support of increased ET-related activity.

A novel aspect of the present study is the demonstration that common functional polymorphisms in genes (*DNASE1*, rs1053874; and *TREX1*, rs11797) that encode DNA-degrading enzymes may influence the ET burden and the composition of inflammatory cells within human coronary thrombi in vivo. Carriers of the *DNASE1* rs1053874 GG genotype exhibited significantly higher densities of MPO- and CD68-positive cells compared with GA/AA carriers, while *TREX1* rs11797 CC genotype carriers displayed increased densities of CitH3-, MPO-, and CD68-positive cells relative to CT/TT carriers. From a biological standpoint, DNase I and TREX1 are deemed to be indispensable for the efficient clearance of extracellular chromatin and DNA fragments released during ETosis. *DNASE1* rs1053874 polymorphism is a well-characterised missense mutation. It has been demonstrated to reduce enzymatic activity and to impair the degradation of extracellular DNA. This, in turn, leads to prolonged persistence of ETs, sustained thrombo-inflammation, larger infarct sizes, impaired microvascular reperfusion, and heightened long-term mortality after acute myocardial infarction [30,31,32,54]. Earlier studies have directly linked this variant to increased susceptibility to myocardial infarction in Japanese populations [32] and to worse clinical outcomes in broader STEMI cohorts [31]. In a similar manner, the *TREX1* rs11797 polymorphism has been demonstrated to influence the function of the three-prime repair exonuclease 1, which is imperative for the degradation of cytosolic DNA and the prevention of aberrant innate immune activation. Impaired TREX1 activity has been associated with defective clearance of DNA, dysregulated type I interferon signalling, the occurrence of autoinflammatory states, and an enhanced thrombotic risk [55]. In the context of STEMI, these genetic variants are likely to exacerbate the potential self-amplifying loop of ETosis by allowing excessive accumulation of prothrombotic ET structures within the culprit coronary thrombus. This process amplifies local inflammation (higher MPO- and CD68-positive cells) and promotes thrombus maturation from fresh to lytic stages. To the best of our knowledge, this is the first in vivo study to establish a link between these specific SNPs and the cellular composition and ET burden of culprit-site coronary thrombi obtained during emergency pPCI. This extends prior associations observed in peripheral blood or post-infarction outcomes to the actual site of thrombosis. This genetic modulation may partly explain inter-individual variability in thrombus progression, reperfusion success, and long-term prognosis highlighting a heritable component in the regulation of local thrombo-inflammation and thrombus cellularity.

From a clinical perspective, the fact that a higher ET load is consistently associated with impaired reperfusion suggests that ETosis may be a promising therapeutic target in STEMI, particularly in patients who present late [21,23,24,25,56,57]. Interventions such as recombinant DNase I or PAD4 inhibitors warrant further investigation in appropriately designed trials.

The findings of this study serve to extend and refine extant knowledge in the field. The presence of ETs in coronary aspirates has been confirmed in previous studies, and a link has been established between higher ET burden and impaired reperfusion as well as clinical events. The novelty of the present work lies in the demonstration of a statistically significant and quantitatively robust increase in CitH3-positive cells in lytic versus fresh thrombi, the documentation of macrophage participation in ETosis within the coronary microenvironment, and the novel identification of associations between *DNASE1* and *TREX1* risk genotypes and heightened densities of inflammatory and ETosis-related cells directly in aspirated human thrombi. The findings of this study provide a more nuanced understanding of the temporal evolution of immunothrombosis and highlight genetically determined inter-individual differences in ET regulation as potential modifiers of thrombus biology and treatment response.

The present study is accompanied by several limitations. The study was conducted in a single centre only and the number of subjects analysed was modest for genetic subgroup analyses. No direct functional assay of DNase I enzymatic activity was performed. It is imperative to note that only cases with sufficient thrombus material were included in the study. Additionally, while immunofluorescence with confocal microscopy would ideally visualise the extracellular, web-like DNA structures decorated with granular proteins (e.g., MPO, NE and calprotectin), practical constraints in this large cohort (*n* = 81) limited its use. The thrombus material was processed as formalin-fixed, paraffin-embedded blocks, which were optimised for standardised immunohistochemistry and high-throughput QuPath quantification. The challenges posed by the study included strong autofluorescence (derivatives of haemoglobin), antigen masking from fixation, and limited sample volume (50–100 mg per patient). These issues rendered reliable multiplex immunofluorescence challenging without compromising primary analyses. However, due to material constraints, the implementation of Western blots for histone degradation or ROS assays was rendered impractical. Future studies employing fresh thrombus material and advanced imaging techniques (e.g., confocal or super-resolution microscopy) will be valuable in further visualising extracellular DNA-MPO/CitH3 webs and CitH3/Cas3 co-localisation, and definitively confirming ETosis morphology.

Nevertheless, the integration of standardised histological thrombus classification, multiplex immunohistochemistry, high-precision QuPath digital image analysis, plasma biomarker profiling, and candidate-gene genotyping offers a unique comprehensive and multi-layered insight into the dynamics of ETs in human coronary thrombosis.

In conclusion, the present findings indicate that ETosis is a significant driver of immunothrombosis, promoting coronary thrombus maturation from the fresh to the lytic stages, sustaining thrombo-inflammation, and contributing to adverse clinical outcomes in patients with STEMI. The time-dependent escalation of this process, the mechanistic interplay with apoptosis and platelets, the association with systemic inflammation, and the modulation by *DNASE1* and *TREX1* functional polymorphisms highlight ETosis as both a pathophysiological cornerstone and a therapeutically actionable target. The targeted modulation of ETs formation or clearance holds considerable potential for enhancing myocardial salvage and improving long-term prognosis in high-risk patients with STEMI. The translation of mechanistic insights into valuable clinical benefit is now possible through larger multicentre studies and randomised controlled trials of ET-directed therapies.

## 4. Materials and Methods

### 4.1. Population and Sample Collection

The present study included patients diagnosed with STEMI according to the European Society of Cardiology guidelines [58], from whom intracoronary thrombus material was aspirated during pPCI. The study was conducted in accordance with the Declaration of Helsinki and approved by the Kaunas Regional Biomedical Research Ethics Committee (approval no. BE-2-74, 22 November 2023). Written informed consent was obtained from each participant. Patients were excluded from participation if they had received thrombolytic therapy within the previous 24 h, had active infection, systemic inflammatory disease, malignancy, end-stage liver or renal failure, or were immunosuppressed.

Peripheral venous blood samples were collected on admission (prior to PCI). All thrombus aspirates were preserved in buffered formalin for a 24 h period and subsequently paraffin-embedded.

The patient characteristics are presented in Table 1.

### 4.2. Histology and Immunohistochemistry

Formalin-fixed, paraffin-embedded tissue blocks obtained from the thrombus aspirate were sectioned at a thickness of 3 µm using a Leica RM2235 rotary microtome (Leica Biosystems, Deer Park, IL, USA), resulting in a total of 20 serial slides for each case. The sections were mounted on Menzel SuperFrost Plus adhesive slides (Menzel, Braunschweig, Germany), air-dried overnight at room temperature, and baked in an oven at 50 °C for at least 12 h to ensure adhesion. Deparaffinisation was performed by immersing the slides in xylene, followed by rehydration through a graded series of ethanol concentrations, and final rinsing in distilled water.

For the purpose of evaluating the age of the thrombus, the slides were subjected to staining using the hematoxylin–eosin and Picro-Mallory methods.

The process of heat-induced epitope retrieval for immunohistochemistry was conducted using the RHS-1 microwave processor (Milestone Medical, Bergamo, Italy). The tissue sections were immersed in a retrieval buffer composed of Tris and EDTA at a pH of 9.0 (Agilent Technologies Inc., Wood Dale, IL, USA, Cat. No. S236784-2) and subjected to heating at 110 °C for a period of eight minutes. Immunohistochemical staining was carried out in Sequenza slide racks with the corresponding cover plates (Shandon Diagnostics Limited, Runcorn, UK). The endogenous peroxidase activity was quenched by incubating the slides with a peroxidase-blocking reagent (Agilent Technologies Inc., Wood Dale, IL, USA, Cat. No. S202386-2) for a period of 15 min. The slides were subsequently subjected to a washing process in a wash buffer (Agilent Technologies Inc., Wood Dale, IL, USA, Cat. No. S300685-2).

Primary antibodies were appropriately diluted with antibody diluent (Agilent Technologies Inc., Wood Dale, IL, USA, Cat. No. S080983-2), as specified in Table 4, and were incubated at room temperature for 60 min.

The sections were processed using the EnVision FLEX+ visualisation system and HRP DAB+ or HRP magenta chromogen (Agilent Dako, Wood Dale, IL, USA, K800221-2, K800921-2, GV92511-2), following the manufacturer’s instructions. Nuclei were counterstained with hematoxylin (Agilent Technologies Inc., Wood Dale, IL, USA, Cat. No. S330930-2). After counterstaining, the sections were dehydrated in an ascending series of ethanol solutions, cleared in xylene, and mounted with a permanent mounting medium.

In order to assess protein co-localisation, a double immunohistochemical staining procedure was performed on tissue sections for CitH3 with MPO. Following antigen retrieval and enzyme blocking (as previously described), sections were first incubated with anti-CitH3 antibody and visualised using the EnVision FLEX+ visualisation system and HRP magenta chromogen (Agilent Dako, Wood Dale, IL, USA, K800221-2, GV92511-2). Subsequently, 0.3 M sulfuric acid was applied. This process was replicated for MPO. Prior to incubation with emerald chromogen (Neo-Biotech, Nanterre, France, NB-23-00153), sections were counterstained with hematoxylin, in accordance with the manufacturer’s protocol. For each case, three consecutive serial sections were prepared: the first was stained for CitH3 only, the second for simultaneous CitH3 and MPO (or Cas3) detection, and the third for MPO alone.

The process of double immunohistochemical staining for CitH3-CD68 was conducted utilising the ImmPRESS Duet Double Staining HRP/AP Polymer Kit (anti-rabbit IgG, green; anti-mouse IgG, magenta; Vector Laboratories, Inc., Newark, CA, USA; Cat. No. MP-7714-15) according to the manufacturer’s instructions.

Antibody specificity was verified using positive control tissue sections (3 µm thick, see Table 4). Negative controls included substituting the primary antibodies with isotype-matched, non-immune IgG at equivalent concentrations and dilutions, resulting in an absence of a specific signal. All staining procedures were performed in parallel and adhered to the best practices established for immunohistochemical reproducibility, including those outlined by expert consensus groups on antibody validation and positive control standardisation in diagnostic immunohistochemistry [59,60].

### 4.3. Image Acquisition and Analysis

The digitisation of the stained tissue slides was performed using a Pannoramic MIDI scanner (3DHistech, Budapest, Hungary) with a Plan-Apochromat 20×/0.8 NA objective and a Hitachi HV-F22CL camera, resulting in a spatial resolution of 0.2325 µm per pixel.

A concomitant evaluation of all stained preparations of coronary thrombi from 94 subjects was conducted using SlideViewer software (version 2.5, 3DHistech, Budapest, Hungary). A total of 81 subjects were included in the final analysis. Eight subjects were excluded from the study because only atherosclerotic plaque material was present in the aspirates, and an additional five subjects were excluded due to insufficient material for analysis. The thrombi were then classified according to their histological age, a process which was based on the well-known histological features of fresh, lytic, and organised thrombi [28] by two researchers independently. The consensus classification was determined by considering all individual samples, and any challenging cases were discussed with a certified pathologist (D.P.), who acted as an independent observer.

Digital whole-slide images of thrombus tissue with CitH3-, MPO-, and CD68-positive cells (DAB chromogen) were analysed using QuPath software (version 0.6.0) [61]. Within each slide 9–12 regions of interest (ROIs) were selected. The cell density (cells/mm^2^) of all cell types was evaluated for each ROI. All analyses were performed without knowledge of the clinical data or thrombus classification. The inter-observer and intra-observer intraclass correlation coefficients for quantitative cell density were both greater than 0.9.

Two independent observers manually annotated all relevant tissue areas using SlideViewer software (version 2.5, 3DHistech, Budapest, Hungary) for semi-quantitative scoring. For double-stained samples of CitH3 (stained with magenta) and MPO (stained with Emerald), the CitH3 immunostaining proportion score was calculated as the proportion of CitH3-positive cells to MPO-positive cells according to the following scoring method: 1—<1%, 2—1–10%, 3—11–33%, 4—34–66%, 5—>66% [62]. Cas3-positive cells were graded using a unified 5-point system that integrated staining intensity and the proportion of positive cells. This approach followed the standardised protocol described in the Human Protein Atlas [63], with the following categories: 0 = not detected (negative or weak staining in less than 25% of cells); 1 = low (weak staining in at least 25% of cells or moderate staining in less than 25% of cells); 2 = medium (moderate staining in at least 25% of cells or strong staining in less than 25% of cells); 3 = high (strong staining of at least 25% but less than 50% of cells); 4 = very high (strong staining of at least 50% of cells). CD61-positive fields were graded using a 4-point system: 1—<25%, 2—26–50%, 3—51–75%, 4—>76% of the total area. The inter-observer and intra-observer variability was evaluated by Kappa (κ) statistics (Cohen’s κ coefficient > 0.9).

To maintain objectivity, all images were deidentified prior to analysis, and quantifications were performed in a blinded manner. Reproducibility was confirmed through the blinded reevaluation of a subset of representative images.

### 4.4. Plasma ET-Related Markers Quantification

The quantification of circulating dsDNA was conducted using a fluorescence-based assay with the Quant-iT PicoGreen dsDNA Kit (Invitrogen/Thermo Fisher Scientific, Waltham, MA, USA, Cat. No. P11496), in accordance with the manufacturer’s instructions. The samples were excited at 480 nm and the fluorescence emission intensity was measured at 520 nm using an Infinite 200 PRO M Plex microplate reader (Tecan Trading, Mannedorf, Switzerland). Subsequently, the results were normalised to the standard curve.

The quantification of plasma MPO, NE and CitH3 was performed using the enzyme-linked immunosorbent assay (ELISA) technique. The measurement of MPO and NE was conducted using the Human Myeloperoxidase Instant ELISA Kit (Invitrogen/Thermo Fisher Scientific, Waltham, MA, USA, Cat. No. BMS2038INST) and the Human PMN (Neutrophil) Elastase ELISA Kit (Invitrogen/Thermo Fisher Scientific, Waltham, MA, USA, Cat. No. BMS269), respectively. CitH3 levels were measured using the Citrullinated Histone H3 (Clone 11D3) ELISA Kit (Cayman Chemical, Ann Arbor, MI, USA) in accordance with the manufacturer’s instructions. ELISAs were performed in duplicate. The optical density was measured using an Infinite 200 PRO M Plex microplate reader (Tecan Trading, Mannedorf, Switzerland) at a wavelength of 450 nm, with the reference wavelength set to 620 nm. The optical density readings were then normalised to a standard curve.

### 4.5. DNA Extraction and Genotyping

Genomic DNA was manually isolated from peripheral blood leukocytes using the PureLink Genomic DNA Mini Kit (Invitrogen/Thermo Fisher Scientific, Waltham, MA, USA, Cat. No. K182002) according to the manufacturer’s instructions. The concentration and purity of the samples were assessed using a Nanodrop 1000 spectrophotometer (Thermo Fisher Scientific, Wilmington, DE, USA). Isolated DNA was stored at a temperature of −20 °C until analysis.

Single-nucleotide polymorphisms of the *DNASE1* (rs1053874) and *TREX1* (rs11797) were evaluated using real-time PCR with TaqMan SNP Genotyping Assays (C___8928399_1, C___11537906_20, respectively) according to the manufacturer’s instructions (Applied Biosystems/Thermo Fisher Scientific, Waltham, MA, USA). The cycling programme commenced with a heating step at 95 °C for a duration of 5 min. This was followed by 40 cycles at 95 °C for 15 s and 60 °C for a period of one minute. Allele-specific fluorescence was analysed on the QuantStudio 5 Real-Time PCR System (Applied Biosystems/Thermo Fisher Scientific, Waltham, MA, USA). Genotyping was successfully performed in 63 patients (Table 5).

### 4.6. Statistical Analysis

Categorical variables are presented as frequencies and percentages. Differences between categorical variables were assessed using the Chi-square test or Fisher’s exact test. The Chi-square goodness-of-fit test was used to evaluate the Hardy–Weinberg equilibrium for genotype distributions.

The normality of continuous variables was assessed using the Shapiro–Wilk test.

Non-normally distributed variables were expressed as medians with interquartile ranges and compared between two independent groups using the Mann–Whitney U test. For the normally distributed variable (cell density), linear mixed-effects models were applied. The data had a three-level hierarchical structure: tissue regions of interest (ROIs, level 1) were nested within patients (level 2), and patients were grouped by thrombus age (fresh vs. lytic), TIMI flow (3 vs. 2/1), *DNASE1* genotype (GG, GA, or AA), and *TREX1* genotype (CC, CT, or TT) (level 3). Patient group was included as a fixed factor. To account for the non-independence of multiple tissue sections from the same patient, random intercepts were specified at the patient level. Models were fitted using restricted maximum likelihood estimation. Denominator degrees of freedom for tests of fixed effects were calculated using the Satterthwaite approximation, which is suitable for unbalanced designs.

When the omnibus test for the fixed effect of *DNASE1* or *TREX1* genotype was statistically significant, pairwise post hoc comparisons between the three genotypes were performed on the estimated marginal means with Bonferroni correction to control the family-wise error rate. Results for cell density are reported as estimated marginal means ± standard error.

Spearman’s rank correlation test was employed to evaluate correlation trends.

A two-sided *p*-value < 0.05 was considered statistically significant. All statistical analyses were performed using IBM SPSS Statistics (Version 31.0, IBM Corp., Armonk, NY, USA).

## 5. Conclusions

This study demonstrates that ETosis, identified by CitH3 positivity, is a consistent feature of coronary thrombi in patients with STEMI undergoing pPCI. Extracellular traps were present in all thrombus aspirates; however, their extent was significantly greater in lytic (older) thrombi compared with fresh thrombi (mean 1348 vs. 591 CitH3-positive cells/mm^2^, *p* < 0.001).

Enhanced ETosis was associated with neutrophil and monocyte/macrophage infiltration (as determined by MPO- and CD68-positive cells), apoptosis (as indicated by the presence of Cas3), prolonged ischemia (time from symptom onset to pPCI), systemic inflammation (elevated NLR and C-reactive protein), and impaired myocardial reperfusion (lower TIMI flow grades) after pPCI.

In addition, functional polymorphism in *DNASE1* (rs1053874) was found to be associated with inflammatory cell accumulation, and *TREX1* (rs11797) was associated with both inflammatory cell accumulation and ETosis burden within the thrombi.

The findings indicate that ETosis increases with coronary thrombus maturation and is associated with intensified local and systemic thrombo-inflammation, as well as worse reperfusion outcomes in STEMI. It can thus be posited that ETosis may represent a potential therapeutic target. The targeted modulation of ETs formation or degradation (e.g., via PAD4 inhibitors or recombinant DNase I) warrants further investigation in larger multicentre studies and randomised controlled trials, particularly in patients with delayed presentation or high-risk genotypes. It is to be acknowledged that further validation of these results and the translation of ET-targeted therapies into improved myocardial salvage and long-term prognosis will require multicentre studies and randomised controlled trials.

## Figures and Tables

**Table 1:** Spearman’s rank correlation coefficients (ρ) between thrombus immunohistochemical components.

Parameter	CitH3 Immuno-Staining Score	Cas3 Immuno-Staining Score	CD61 Immuno-Staining Score	CitH3-Positive Cells/mm^2^	MPO-Positive Cells/mm^2^
*Fresh thrombi (n = 41)*					
Cas3 immunostaining score	0.500 **				
CD61 immunostaining score	0.195	−0.032			
CitH3-positive cells/mm^2^	0.693 **	0.561 **	−0.061		
MPO-positive cells/mm^2^	0.444 **	0.422 **	−0.127	0.548 **	
CD68-positive cells/mm^2^	0.321	0.241	0.417 *	0.239	0.592 **
*Lytic thrombi (n = 40)*					
Cas3 immunostaining score	0.313				
CD61 immunostaining score	0.260	−0.072			
CitH3-positive cells/mm^2^	0.662 **	0.364 *	0.216		
MPO-positive cells/mm^2^	0.166	−0.001	0.303	0.558 **	
CD68-positive cells/mm^2^	−0.172	−0.250	0.406 *	−0.115	0.265

* *p* < 0.05 (2-tailed); ** *p* < 0.01 (2-tailed). Abbreviations: Cas3, caspase 3; CD, cluster of differentiation; CitH3, citrullinated histone H3; MPO, myeloperoxidase. Significant *p* values are presented in bold.

**Table 2:** Characteristics of included patients.

Parameter	All Patients *n* = 81	Fresh Thrombi *n* = 41	Lytic Thrombi *n* = 40	*p* Value
Age, yrs	64.19 (13.1)	62.2 (13.1)	66.2 (13.0)	0.173
Male, *n* (%)	58 (71.60)	29 (70.73)	29 (72.50)	0.860
Smoking, *n* (%)	26 (32.10)	13 (31.71)	13 (32.50)	0.939
Hypertension, *n* (%)	70 (86.42)	34 (82.93)	36 (90.00)	0.353
Time from symptoms onset to pPCI, h	3.70 [2.56–10.48]	3.38 [2.27–8.56]	5.29 [3.08–13.52]	**0.047**
*Infarct-related artery:*				0.596
Left anterior descending, *n* (%)	31 (38.27)	14 (34.14)	17 (42.5)	
Circumflex, *n* (%)	6 (7.41)	4 (9.76)	2 (5.0)	
Right coronary artery, *n* (%)	44 (54.32)	23 (56.10)	21 (52.5)	
*TIMI flow grade after pPCI:*				0.934
1, *n* (%)	2 (2.47)	1 (2.44)	1 (2.50)	
2, *n* (%)	11 (13.58)	5 (12.20)	6 (15.00)	
3, *n* (%)	68 (83.95)	35 (85.36)	33 (82.50)	
In-hospital death	6	4	2	
*Laboratory data at admission:*				
White blood cells count, ×10^9^/L	12.00 (3.83)	11.37 (3.91)	12.64 (3.69)	0.138
Neutrophils count, ×10^9^/L	8.94 (3.72)	8.26 (3.96)	9.64 (3.37)	0.096
Lymphocytes count, ×10^9^/L	1.70 [1.15–2.70]	2.10 [1.45–3.00]	1.40 [1.00–2.18]	**0.012**
NLR	4.91 [2.65–8.28]	4.07 [1.96–6.37]	5.82 [3.72–10.26]	**0.009**
Platelets count, ×10^9^/L	241.33 (60.68)	248.30 (65.09)	234.18 (55.73)	0.304
Prothrombin time, s	24.85 [23.05–26.38]	24.45 [22.88–26.00]	25.35 [23.35–26.93]	0.236
Prothrombin activity, %	87.87 (19.62)	90.24 (20.45)	84.87 (18.41)	0.266
C-reactive protein, mg/L	5.50 [5.00–20.70]	5.00 [5.00–10.05]	8.80 [5.00–36.30]	**0.033**
hs Troponin I, ng/L	2152.8 [35.50–34,458.65]	196.9 [22.85–21,248.20]	7036.95 [46.02–46,308.33]	0.162

The data are presented as mean (standard deviation) or median [interquartile range, IQR], depending on the distribution of the variable. Categorical variables are displayed as counts and percentages. Abbreviations: hs, high sensitivity; NLR, neutrophil–lymphocyte ratio; pPCI, primary percutaneous coronary intervention; TIMI, Thrombolysis in Myocardial Infarction flow grade: 0—no perfusion; 1—penetration without perfusion; 2—partial perfusion; 3—complete perfusion. Significant *p* values are presented in bold.

**Table 3:** Spearman’s rank correlation coefficients (ρ) between plasma ETs markers, admission laboratory parameters, time from symptoms onset to pPCI, and thrombus immunohistochemical components.

**Parameter**	**CitH3 Immunostaining Score**	**Cas3 Immunostaining Score**	**CD61 Immunostaining Score**	**CitH3-Positive Cells/mm^2^**	**MPO-Positive Cells/mm^2^**	**CD68-Positive Cells/mm^2^**
*Fresh thrombi (n = 41)*						
Plasma CitH3, ng/mL	0.299	0.102	−0.049	0.066	−0.241	−0.184
Plasma dsDNA, ng/mL	−0.089	0.215	0.035	0.037	0.100	0.413
Plasma MPO, ng/mL	0.194	0.100	−0.103	−0.045	−0.107	−0.291
Plasma NE, ng/mL	0.242	0.206	−0.053	0.546 **	−0.073	−0.197
White blood cells, ×10^9^/L	0.245	0.283	0.165	0.382 *	0.249	0.071
Neutrophils, ×10^9^/L	0.265	0.258	0.066	0.411 *	0.304	0.083
Lymphocytes, ×10^9^/L	−0.020	0.093	0.087	−0.204	−0.172	−0.198
NLR	0.157	0.093	0.047	0.386 *	0.260	0.192
Platelets, ×10^9^/L	0.001	−0.059	0.098	0.016	−0.137	−0.266
Prothrombin time, s	0.046	−0.185	−0.192	−0.020	0.150	0.127
Prothrombin activity, %	−0.010	0.217	0.185	0.057	−0.129	−0.117
C-reactive protein, mg/L	0.182	0.098	0.019	0.156	0.014	−0.148
hs Troponin I, ng/L	0.107	−0.008	−0.044	0.132	0.001	−0.017
Time from symptoms onset to pPCI, h	0.195	0.303	−0.165	0.232	0.285	0.141
*Lytic thrombi (n = 40)*						
Plasma CitH3, ng/mL	0.152	0.110	0.224	0.104	0.200	0.249
Plasma dsDNA, ng/mL	−0.272	−0.065	0.098	−0.078	0.308	0.073
Plasma MPO, ng/mL	0.206	0.463 **	−0.008	0.112	0.068	−0.086
Plasma NE, ng/mL	−0.186	−0.005	0.198	0.051	0.322	0.366
White blood cells, ×10^9^/L	−0.285	−0.445 **	0.153	−0.091	0.444 **	0.097
Neutrophils, ×10^9^/L	−0.324 *	−0.410 **	0.163	−0.133	0.484 **	0.068
Lymphocytes, ×10^9^/L	0.132	−0.166	−0.083	0.125	−0.271	−0.118
NLR	−0.246	−0.079	0.124	−0.186	0.434 **	0.106
Platelets, ×10^9^/L	−0.094	0.072	−0.239	0.065	0.158	−0.152
Prothrombin time, s	0.262	0.392 *	0.106	0.285	0.141	−0.064
Prothrombin activity, %	−0.240	−0.379 *	−0.125	−0.244	−0.106	0.030
C-reactive protein, mg/L	−0.092	0.031	0.299	0.188	0.492 **	0.221
hs Troponin I, ng/L	0.074	0.487 **	0.023	0.065	0.276	0.104
Time from symptoms onset to pPCI, h	0.027	0.090	−0.001	0.274	0.536 **	0.141

* *p* < 0.05 (2-tailed); ** *p* < 0.01 (2-tailed). Abbreviations: Cas3, caspase 3; CD, cluster of differentiation; dsDNA, double-stranded DNA; CitH3, citrullinated histone H3; hs, high sensitivity; MPO, myeloperoxidase; NE, neutrophil elastase; NLR, neutrophil–lymphocyte ratio. Significant *p* values are presented in bold.

**Table 4:** Characteristics of primary antibodies used for immunohistochemistry.

Antibody	Species (Immunogen)	Dilution	Manufacturer (Catalog Number)	PRID	Lot Number	Positive Control Tissue
CitH3 (citrulline R2 + R8 + R17)	Rabbit polyclonal (ChIP Grade)	1:1000	Abcam, Cambridge, UK (ab5103)	AB_304752	1091694-1	Appendix (acute appendicitis)
Cas3	Rabbit polyclonal	1:1000	Merck (Sigma-Aldrich), Darmstadt, Germany (HPA002643)	AB_1846048	B117459	Tonsil with germinal centres
MPO	Mouse monoclonal, 59A5	BOND RTU Pr	Leica Biosystems, Deer Park, IL, USA (PA0491)	AB_10555993	79205396	Spleen, appendix (acute appendicitis)
CD68	Mouse monoclonal, 514H12	1:100	Leica Biosystems, Deer Park, IL, USA (NCL-L-CD68)	AB_563622	6131699	Brain, tonsil
CD61 (GPIIIa)	Mouse monoclonal, 2F2	BOND RTU Pr	Leica Biosystems, Deer Park, IL, USA (PA0308)	AB_564092	83305	Bone marrow, internal
CD34	Mouse monoclonal, QBEnd 10 (Endothelial cell membranes obtained as vesicles from human placenta)	1:100	Agilent Dako, Wood Dale, IL, USA (M7165)	AB_2063006	41869479	Appendix, liver
αSMA	Mouse monoclonal, cl 1A4 (N-terminal synthetic decapeptide of αSMA)	1:100	Agilent Dako, Wood Dale, IL, USA (M085101-2)	AB_2223500	41480058	Appendix, liver

Abbreviations: αSMA, alpha smooth muscle actin; CD, cluster of differentiation; MPO, myeloperoxidase; Cas3, caspase 3; CitH3, citrullinated histone H3.

**Table 5:** Genotype and allele frequencies of *DNASE1* rs1053874 and *TREX1* rs11797 in the study group.

*DNASE1*, rs1053874 (NM_005223.4:c.731G>A, p.Arg244Gln)		*TREX1*, *rs11797* (NM_033629.6:c.531T>C, p.Tyr177=)	
	Genotype		
GG	26 (41.3)	CC	18 (28.6)
GA	31 (49.2)	CT	24 (38.1)
AA	6 (9.5)	TT	21 (33.3)
Total	63 (100)	Total	63 (100)
	Alleles		
G	83 (0.659)	C	60 (0.476)
A	43 (0.341	T	66 (0.524)
Total	126 (1.00)	Total	126 (1.00)
	HW eq		
χ^2^	0.562		3.520
*p*	0.454		0.061

Abbreviations: HW eq, Hardy–Weinberg equilibrium.

## Data Availability

The original contributions presented in this study are included in the article/Supplementary Materials. Further inquiries can be directed to the corresponding author.

## Supplementary Materials

The following supporting information can be downloaded at: https://www.mdpi.com/article/10.3390/ijms27135998/s1.

## Abbreviations

The following abbreviations are used in this manuscript:

αSMA	Alpha smooth muscle actin
Cas3	Caspase 3
CitH3	Citrullinated histone H3
CD	Cluster of differentiation
DAB	Diaminobenzidine
dsDNA	Double-stranded DNA
DNase I	Deoxyribonuclease 1
IQR	Interquartile range
MPO	Myeloperoxidase
NE	Neutrophil elastase
NLR	Neutrophil-lymphocyte ratio
PAD4	Peptidylarginine deiminase 4
pPCI	Primary percutaneous coronary intervention
ROI	Regions of interest
SNP	Single nucleotide polymorphism
TIMI	Thrombolysis in Myocardial Infarction flow grade
TREX1	Three-prime repair exonuclease 1
VIF	Variance inflation factor
