# Supplementary material for: Extracellular Traps in Coronary Thrombus Aspirates from Patients with ST-Elevation Myocardial Infarction
Source: Int J Mol Sci. 2026 Jul 3;27(13):5998. doi: 10.3390/ijms27135998 (PMC13361969; doi:10.3390/ijms27135998)
Supplement: Supplementary file 1 [file ijms-27-05998-s001.zip › ijms-4375381-supplementary.pdf]

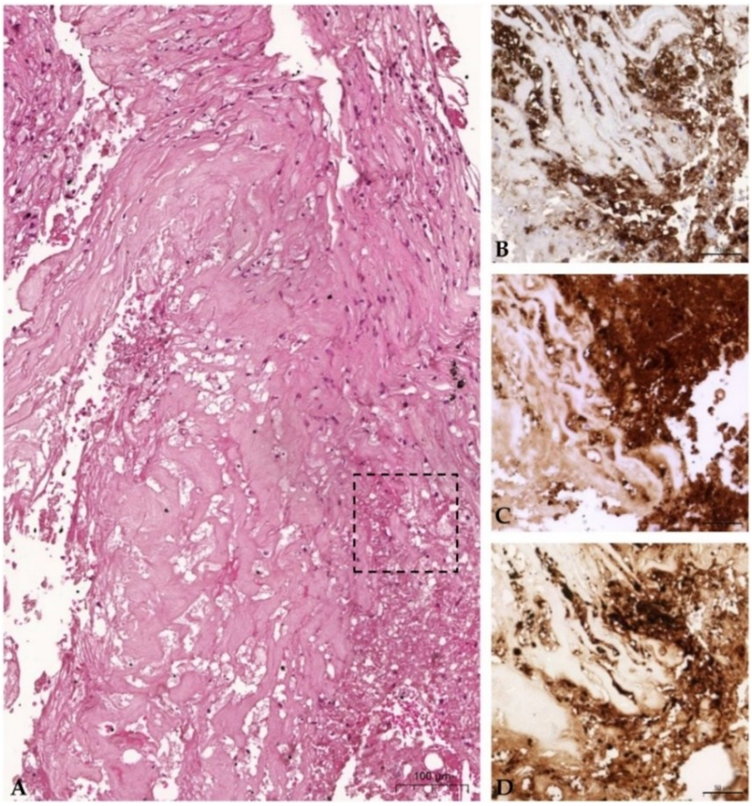


**Figure S1.** Representative images of coronary artery material extracted via thrombus aspiration from patients with ST-segment elevation myocardial infarction show atherosclerotic plaque (A, HE stain) with macrophages (foam cells), which are positive for CD68 (B), MPO (C) and CitH3 (D); immunohistochemical stain with DAB chromogen (brown). Scale bar: 100 μm (A) and 50 μm (B, C, D). Images were captured from digital slides with an original magnification of 20x. Abbreviations: HE, hematoxylin and eosin stain; CD, cluster of differentiation; MPO, myeloperoxidase; CitH3, citrullinated histone H3.

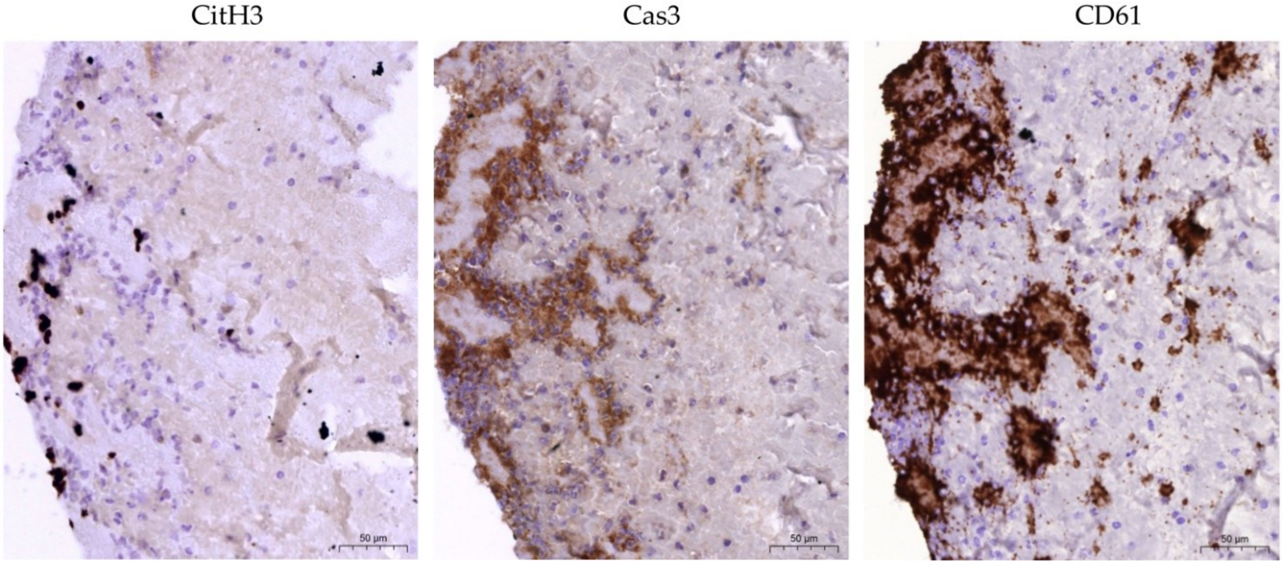


**Figure S2**. Localisation of immunohistochemical expression of CitH3, Cas3, and CD61. Scale bar: 50 μm. Images were captured from digital slides with an original magnification of 20x. Abbreviations: Cas 3, caspase 3; CD, cluster of differentiation; CitH3, citrullinated histone H3.

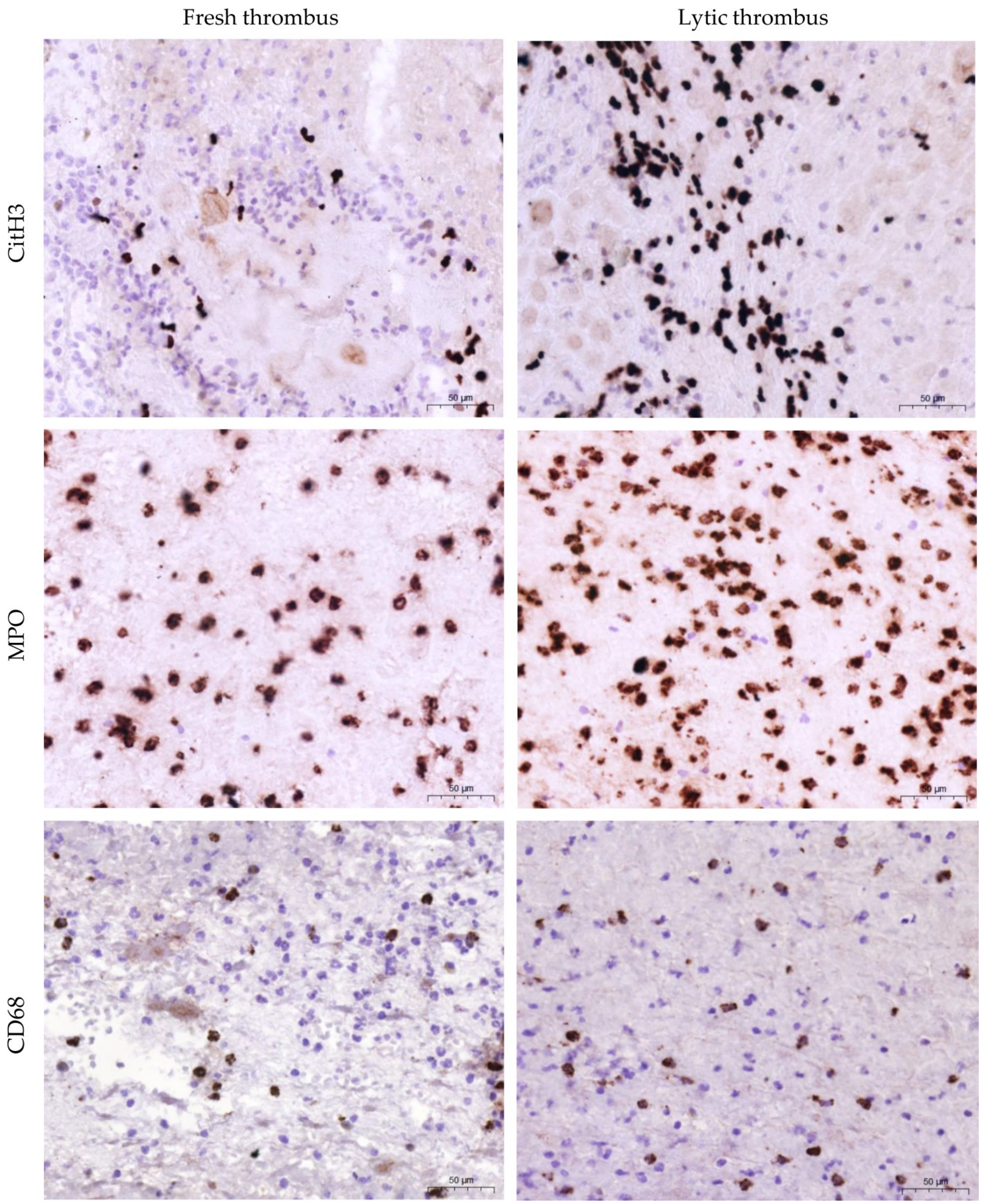


**Figure S3.** Representative images of slides stained for immunohistochemistry, ready for digital image analysis. Scale bar: 50 µm. Images were captured from digital slides with an original magnification of 20x. Abbreviations: Cas3, caspase 3; CD, cluster of differentiation; MPO, myeloperoxidase.

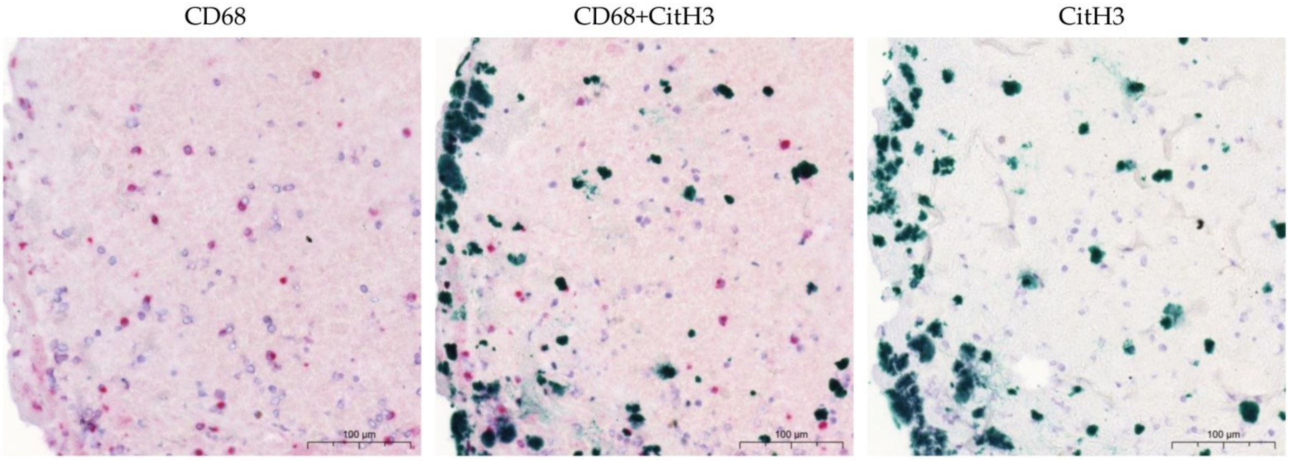


**Figure S4.** Comparative immunohistochemical expression of CitH3 (stained in green) and CD68 (stained in red). Scale bar: 100 µm. Images were captured from digital slides with an original magnification of 20x. Abbreviations: CD, cluster of differentiation; CitH3, citrullinated histone H3.

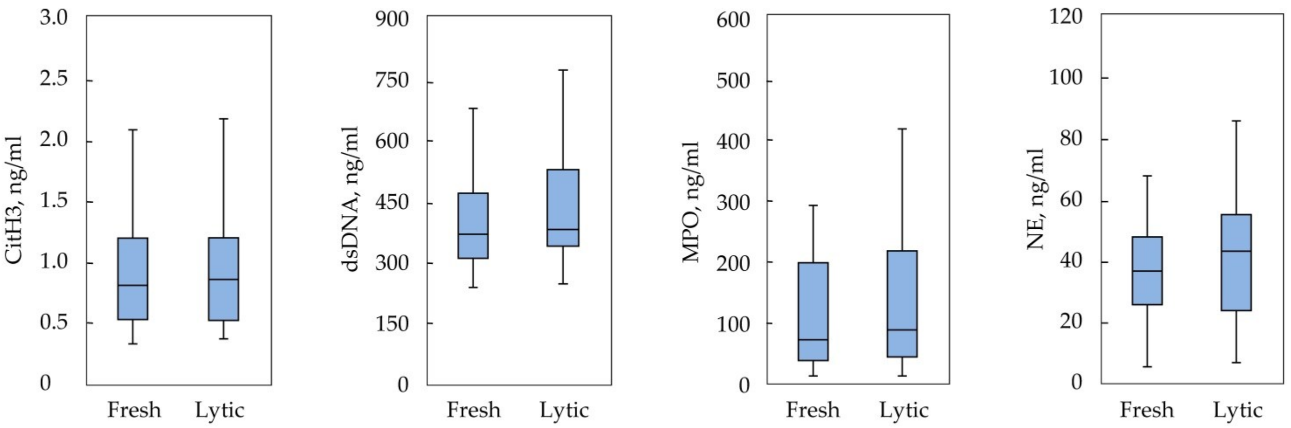


**Figure S5.** Levels of plasma NETs markers in fresh (n = 41) and lytic (n = 40) thrombus groups. Data are presented as median, the 25th and 75th percentiles, and minimum and maximum values (whiskers); the Mann–Whitney U test was used. Abbreviations: CitH3, citrullinated histone H3; ds DNA, double-stranded DNA; MPO, myeloperoxidase; NE, neutrophil elastase.
